# Remodeling the Medication Collection Process With Prescription in Locker Box (PILBOX): Prospective Cross-sectional Study

**DOI:** 10.2196/23266

**Published:** 2022-06-27

**Authors:** Christina Lim Jit Fan, Goh Boon Kwang, Vivian Chee Wing Ling, Tang Woh Peng, Bandy Goh Qiuling

**Affiliations:** 1 Department of Allied Health (Pharmacy) SingHealth Polyclinics Singapore Singapore

**Keywords:** PILBOX, medication collection process, dispensing, remodeling, locker box, medication, pharmaceuticals, prescription

## Abstract

**Background:**

Traditionally, patients wishing to obtain their prescription medications have had to physically go to pharmacy counters and collect their medications via face-to-face interactions with pharmacy staff. Prescription in Locker Box (PILBOX) is a new innovation allowing patients and their caregivers to collect medication asynchronously, 24/7 at their convenience, from medication lockers instead of from pharmacy staff.

**Objective:**

This study aimed to determine the willingness of patients and caregivers to use this new innovation and factors that affect their willingness.

**Methods:**

This prospective cross-sectional study was conducted over 2 months at 2 public primary health care centers in Singapore. Patients or caregivers aged 21 years and older who came to pharmacies to collect medications were administered a 3-part questionnaire face-to-face by trained study team members after they gave their consent to participate in the study.

**Results:**

A total of 222 participants completed the study. About 40% (89/222, 40.1%) of participants were willing to use PILBOX to collect their medications. Among participants who were keen to use the PILBOX service, slightly more than half (47/89, 53%) were willing to pay for the PILBOX service. Participants felt that ease of use (3.5 [SD 1.2]) of PILBOX was the most important factor affecting their willingness to use the medication pickup service. This was followed by waiting time (3.4 [SD 1.3]), cost of using the medication pickup service (3.0 [SD 1.4]), and 24/7 accessibility (2.6 [SD 1.4]). This study also found that age (*P*=.01), language literacy (*P*<.001), education level (*P*<.001), working status (*P*=.01), and personal monthly income (*P*=.01) were factors affecting the willingness of patients or caregivers to use PILBOX.

**Conclusions:**

Patients and caregivers are keen to use PILBOX to collect their medications for its convenience and the opportunity to save time if it is easy to use and not costly.

## Introduction

Many health care systems in the world require patients to physically go to the dispensary or pharmacy to refill or collect their medications. This practice has been ongoing across the globe for a long time [1,2].

Dispensing medications to patients usually encompasses the provision of some basic information about the medication such as the indication, dose, frequency, and duration [3,4]. The face-to-face encounter at the dispensary or pharmacy to collect the medications provides an opportunity for patients to be counseled on the use of their medications and have their concerns pertaining to their medications addressed [5].

This long-standing practice prevails because health care providers and patients instinctively associate the collection of medications by patients as constituting a natural end to their visit to the clinic [6]. A problem may arise if patients are faced with time constraints and cannot afford the extended time waiting for their turn to see the doctor followed by waiting to collect their medications. This problem is especially aggravated for polyclinic patients due to the high volume of patients and resulting long wait times for patients. The inconvenience caused by having to physically collect the prescribed medications and the long wait time the process entails have also contributed to a high medication nonadherence rate worldwide [7].

If one were to adopt a fresh pair of lenses, a visit to the doctor needs not be irreversibly coupled with the medication collection process. It was this out-of-the-box thinking that inspired the study team to envision the development of an innovation that would decouple the medication collection process from the doctor’s visit and allow the patient a choice of collecting their prescribed medication at a separate convenient occasion after having visited their doctor for consultation.

Telecommunications and technological advances such as the invention of the phone, broadband, internet, and mobile apps are potentially game-changers in these modern times [8-10]. They enable alternative medication refill or collection modes to be made available to patients [2]. An example of such an alternative is the delivery of medications to patients via courier service or mail-order pharmacy to their preferred delivery locations with a few button clicks or a phone call to the dispensary or pharmacy.

However, not all alternative medication refill or collection modes are feasible to implement, and some may not prove attractive to patients or their caregivers [11-13]. Some solutions are costly to implement and may require dispensaries or pharmacies to pass on some or all of the implementation and operational costs to users (patients or caregivers) [11] . Others may generate new problems or inconveniences to users such as requiring users to block off a long stretch of their valuable and limited time to await the delivery [13]. Improper handling of the delivered medications by the courier personnel may also have an adverse impact on medication safety [14]. Medication safety may present a significant problem to successful implementation of these alternative medication refill or collection modes, particularly in countries with underdeveloped transport networks and infrastructures [15]. In such countries, the ability of courier personnel to safeguard the quality of the medications during transit may not be a given, even with their best efforts.

The current health care system in Singapore, like many countries worldwide, requires patients to collect their medications physically at the hospital or clinic dispensaries or retail pharmacies. Singapore’s health care services are provided by both private health care providers and public health care institutions. Although there are established medication delivery services run by private operators, patients may not always be prepared to set aside a few hours to wait for the delivery of their medications as they cope with long work hours and many other competing commitments in fast-paced Singapore, such as running errands and taking children to and from classes.

Thus we have envisioned and developed Prescription in Locker Box (PILBOX) as a new innovation to fill the gap. This new innovation is a first in Singapore. The idea is simple. Rather than requiring users to stay in a given location for a long stretch of time to wait for medications to be delivered to them, why not deposit the medications in a secure location for users to pick up at their convenience? Hence, the PILBOX was conceptualized and developed.

The PILBOX station consists of a few columns of lockers of different sizes with one single attached console that allows users to input a unique access code to retrieve their medication parcels from their allocated locker. The locker station is equipped with a security camera, autolock mechanism, and lock-down system to ensure the medication parcels are safely kept until their collection by their rightful owners. The security camera will deter people with malicious intent from targeting the lockers and the medications contained within. Once a medication parcel has been collected, the locker will be autolocked to ensure the locker is inaccessible to unauthorized personnel. Further, the station lock-down system will prevent power outage situations from triggering the unintended opening of lockers and unauthorized retrieval of medication parcels.

The locker station maintains an ambient temperature (ie, at or below 25 ℃ [77 ℉]) due to 24/7 air conditioning of the room housing the station. To ensure the medications will be stored at the suitable temperature until retrieval by the user, ambient environment temperature is monitored continuously using temperature loggers. This is important to preserve the quality of the medications until collection by users.

Suitable lighting has also been installed in the locker station to facilitate easy retrieval of medications. Further, the PILBOX is equipped with an information technology (IT) system that allows easy tracking of use of the locker system and booking of the lockers for collection of medications by users. It can also generate an audit trail to ensure accountability of each occasion of access to the lockers.

As PILBOX is a new innovation in Singapore, a study was conducted to determine the willingness of patients and caregivers to use the medication pickup service and factors that may affect their use. The information gleaned will allow us to develop appropriate strategies to promote the use of the PILBOX service to patients and increase adoption. The study also aimed to test the following hypotheses:

- Participants who are willing to use PILBOX are willing to pay for the medication pickup service.
- Participants are more willing to use the PILBOX if their wait time at the pharmacy is long.
- Participants are more willing to use PILBOX if they are not satisfied with their wait time at the pharmacy.
- Participants are more willing to use PILBOX if they refill their prescriptions or collect medications from the pharmacy frequently.
- Participants are more willing to use PILBOX if the traveling time to the PILBOX (located within the polyclinic) is less.

## Methods

### Study Design

The prospective cross-sectional study was conducted from December 2015 to January 2016. Participants were convenience sampled at 2 polyclinics situated in the eastern part of Singapore. Polyclinics are public primary health care centers that provide medical care for acute and chronic conditions and provide services such as pharmacy, vaccinations, and health screenings. Prior to the development of PILBOX, patients who needed to collect their prescribed medications or who had prescriptions to refill were required to physically go to the polyclinics’ respective in-house pharmacies where they would be served by pharmacy staff at counters.

### Study Participants and Ethical Approval

The recruited participants were aged 21 years and older. Patients or caregivers visiting the in-house pharmacies at the study sites to fill or refill their prescriptions were invited to participate in the study. Potential participants who could not understand or converse in English or Mandarin were excluded from the study. This study was exempted from ethics review by SingHealth centralized institutional review board (SHP 2015/3035).

Using α=.05, a power of 80%, and an expected correlation coefficient of .30, a calculated sample size of at least 85 participants was required to test the hypotheses on willingness to use PILBOX against their (1) willingness to pay to use the service, (2) wait time at the pharmacy, (3) satisfaction with their wait time at the pharmacy, (4) frequency of prescription refills or medication collections at the pharmacy, and (5) traveling time to the PILBOX.

### Study Instrument

A structured questionnaire developed by the study team was administered face-to-face by trained study team members in a standardized manner (Multimedia Appendix 1). Each participant completed the questionnaire once. The questionnaire solicited information on the following:

- Demographics (ie, age, sex, language literacy, education level, working status, housing type, and personal monthly income)
- Relevant factors that might affect participant satisfaction with pharmacy services (ie, estimated traveling time to polyclinic, frequency of prescription refills and medication collections at the pharmacy, average waiting time at the pharmacy, and satisfaction with waiting time at the pharmacy)
- Willingness to use PILBOX and pay for its use

A 5-point Likert scale (1=not keen, 5=very keen) was used to assess willingness of the participants to use PILBOX.

### Statistical Methods

Chi-square or Fisher exact tests were used to determine the factors that might affect the willingness of participants to use PILBOX. Pearson correlation tests were used to determine whether participant willingness to use PILBOX correlated with their (1) willingness to pay to use the service, (2) average waiting times at the pharmacy, (3) satisfaction with their waiting times at the pharmacy, and (4) frequency of prescription refills or medication collections at the pharmacy. All analyses were performed using SPSS (version 25.0, IBM Corp) at the 5% significance level.

## Results

### Participant Characteristics

A total of 222 participants completed the study, with 109 females, 101 males, and 12 unspecified. Among the participants, about half (109/222, 49.1%) were persons aged 65 years and older. Almost 1 in 10 participants (19/222, 8.6%) had no formal education, and 1 in 5 (46/222, 20.7%) had primary education. Almost half (104/222, 46.9%) were not working (Table 1).

**Table 1:** Demographics of participants (n=222).

Characteristics			Value, n (%)
**Age group, years (older adult vs adult)**			
	≥65	109 (49.1)	
	<65	99 (44.6)	
	Unspecified	14 (6.3)	
**Age (years)**			
	21-40	12 (5.8)	
	41-64	87 (41.8)	
	≥65	109 (52.4)	
**Sex**			
	Female	109 (49.1)	
	Male	101 (45.5)	
	Unspecified	12 (5.4)	
**Language literacy (self-reported)**			
	English	124 (40.0)	
	Chinese	132 (42.6)	
	Malay	20 (6.5)	
	Tamil	3 (1.0)	
	Other	2 (0.6)	
	Illiterate	14 (4.5)	
	Unspecified	15 (4.8)	
**Education level**			
	No formal education	19 (8.6)	
	Primary	46 (20.7)	
	Secondary	72 (32.4)	
	Above secondary	67 (30.2)	
	Unspecified	18 (8.1)	
**Working status**			
	Working	100 (45.0)	
	Not working	104 (46.9)	
	Unspecified	18 (8.1)	
**Housing type**			
	Public housing	165 (74.3)	
	Private housing	29 (13.1)	
	Other	8 (3.6)	
	Unspecified	20 (9.0)	
**Personal monthly income (S$)**			
	No income	106 (47.7)	
	≤3000	63 (28.4)	
	3001-6000	21 (9.5)	
	>6000	7 (3.2)	
	Unspecified	25 (11.3)	

### Relevant Factors That May Affect Participant Satisfaction With Pharmacy Services

Among the participants who collected their medications in installments, about half (65/150, 43.3%) refilled their prescriptions or collected their medications every 3 months. About 30% (41/150, 27.3%) refilled their prescriptions or collected their medications more than 4 times a year. The median traveling time among the participants was 15 to 30 minutes. Slightly more than half (116/222, 52.3%) of the participants were satisfied with the waiting time at the pharmacy to collect or refill their prescriptions. The median time participants spent at the pharmacies waiting to collect or refill their prescriptions was 31 to 45 minutes (Table 2).

**Table 2:** Relevant factors that may affect participant satisfaction with pharmacy services.

Factors			Value, n (%)
**Estimated traveling time to polyclinic (minutes)**			
	<15	92 (41.4)	
	15-30	63 (28.4)	
	31-45	37 (16.7)	
	>45	13 (5.8)	
	Unspecified	17 (7.7)	
**Collection of medication in installments?**			
	Yes	150 (67.6)	
	No	72 (32.4)	
**Frequency of prescription refills (for patients who collect in installments)**			
	>1 time per month	2 (1.4)	
	Once every month	23 (15.3)	
	Once every 2 months	16 (10.7)	
	Once every 3 months	65 (43.3)	
	Once every 4 months and longer	44 (29.3)	
**Average waiting time at pharmacy (minutes)**			
	<15	16 (7.2)	
	15-30	72 (32.9)	
	31-45	78 (35.1)	
	>45	55 (24.8)	
**Satisfaction with waiting time at pharmacy**			
	Dissatisfied	68 (30.6)	
	Neutral	37 (16.7)	
	Satisfied	116 (52.3)	
	Unspecified	1 (0.4)	
**Willingness to use PILBOX**			
	Not keen at all	65 (29.3)	
	Not keen	25 (11.2)	
	Neutral	37 (16.7)	
	Keen	47 (21.2)	
	Very keen	42 (18.9)	
	Unspecified	6 (2.7)	
**Willingness to pay to use PILBOX^a^**			
	Willing to pay	73 (32.9)	
	Not willing to pay	123 (55.4)	
	Unspecified	26 (11.7)	
**Amount willing to pay to use PILBOX (**S$**)**			
	≥2	55 (75.3)	
	2-5	13 (17.8)	
	5-10	4 (5.5)	
	<10	1 (1.4)	

^a^PILBOX: Prescription in a Locker Box.

### Willingness to Use PILBOX and Willingness to Pay for the Service

About 40% (89/222, 40.1%) of participants were willing to use PILBOX to collect their medications. A similar proportion (90/222, 40.5%) were not willing to, with the rest (37/222, 16.7%) not sure if they would be keen to do so. More than half (123/222, 55.4%) of the participants were not willing to pay for the service, and about a third (73/222, 32.9%) were willing to pay to use PILBOX. Among those who were willing to pay, the majority (55/73, 75%) preferred to pay S$2 (US $1.50) or less to use PILBOX. In Singapore, this amount can pay for a single-direction bus trip or the fee for courier delivery of online purchases, including ordering of food. It also equates to roughly 5% of a typical polyclinic patient’s medication bill. Among the participants who were keen to use PILBOX, slightly more than half (47/89, 53%) were willing to pay for PILBOX (Table 2).

Participants who were not keen to use the medication pickup service were hesitant because they were worried that they could not understand the directions to use the locker station. Concerns over not being able to read and understand the directions for use as indicated on the locker station because of literacy limitations and poor eyesight were reported by participants. Some participants felt that they would need assistance to guide them on the use of the locker station but were at the same time concerned that they might hold up the queue. This deterred them from using the service. A small percentage (3/222, 1.4%) did not own a cellphone, and 3.6% (8/222) felt they were not tech savvy enough to be able to use the automated collection system. Some participants were concerned that the payment kiosks at the locker station would not be able to support the use of their medical benefit cards.

A common reason cited by participants was that they did not mind waiting at the pharmacy for their medications as they were already accustomed to waiting for their medications. Some participants did not feel safe collecting their medications from the locker station because they were worried they might get the wrong medications or wrong quantities of the medications from the lockers. Some preferred to collect their medications in person at the pharmacy as they appreciated the availability of pharmacy staff to address any medication queries or concerns they might have. They also wanted someone to explain the use of their medications to them in person.

On the other hand, reasons motivating participants to use PILBOX include no waiting time at the pharmacy (which would save time for busy people) and being able to collect their medications at a time convenient to them. The medication pickup service would presumably appeal more to tech savvy individuals, as suggested by one participant in the survey.

### Factors Affecting Willingness to Use PILBOX

The participants felt that the ease of use (3.5 [SD 1.2]) of PILBOX was the most important factor affecting their willingness to use the medication pickup service. This was followed by no wait time (3.4 [SD 1.3]), cost of using the medication pickup service (3.0 [SD 1.4]), and 24/7 accessibility (2.6 [SD1.4]). Participants ranked the location of the locker station (2.6 [SD 1.5]) as the least important among 5 factors that might affect their willingness to use the medication pickup service.

This study found that age (*P*=.01), literacy (*P*<.001), education level (*P*<.001), working status (*P*=.01), and personal monthly income (*P*=.01) were factors affecting the willingness of the patients or caregivers to use PILBOX. The same list of factors was also found to be associated with their willingness to pay for use of the medication pickup service (Table 3).

**Table 3:** Factors affecting willingness to use and willingness to pay to use PILBOX.

Factors			Willingness to use PILBOX^a^					*P* value			Willingness to pay to use PILBOX					*P* value
			Not willing		Willing					Not willing			Willing			
**Age (years), n**			—^b^		—		.01			—			—		<.001	
	≥65	54		36		—			76			26		—		
	<65	31		49		—			44			47		—		
**Sex, n**			—		—		.11			—			—		.72	
	Female	49		39		—			64			37		—		
	Male	36		47		—			56			36		—		
**Language literacy (self-reported), n**			—		—		<.001			—			—		—	
	Non-English	54		18		<.001			61			15		<.001		
	English	30		68		<.001			59			58		<.001		
	Illiterate in any language	14		0		<.001			11			0		.01		
	Literate in at least 1 language	70		86		<.001			109			73		.01		
**Education level, n**			—		—		<.001			—			—		<.001	
	Have education	65		84		—			103			73		—		
	No formal education	18		0		—			16			0		—		
	Primary and below	45		10		—			44			12		—		
	Secondary and above	38		74		—			75			61		—		
**Working status, n**			—		—		.01			—			—		<.001	
	Not working	51		35		—			72			27		—		
	Working	32		49		—			47			46		—		
**Housing types, n**			—		—		.08			—			—		.17	
	Private housing	7		16		—			14			14		—		
	Public housing	75		67		—			103			59		—		
**Personal monthly income, n**			—		—		.01			—			—		<.001	
	Have income	29		46		—			43			43		—		
	No income	52		36		—			73			28		—		
**Frequency of medication collection (per year), n**			—		—		.79			—			—		.17	
	≤4	60		25		—			69			32		—		
	>4	7		17		—			23			18		—		
**Traveling Time to Polyclinic (minutes), n**			—		—		.40			—			—		.94	
	≤30	38		32		—			91			55		—		
	>30	16		9		—			29			18		—		

^a^PILBOX: Prescription in Locker Box.

^b^Not applicable.

There was weak positive correlation between participant willingness to use PILBOX and their willingness to pay for use of the medication pickup service (159/222, *r*=.23, *P*<.001). Participant willingness to use PILBOX correlated weakly and negatively with waiting time at the pharmacy (180/222, *r*=–.23, *P*<.001). There was very weak negative correlation between participant willingness to use PILBOX and their satisfaction level with waiting time at the pharmacy (180/222, *r*=–.06, *P*=.47). On the other hand, there was very weak positive correlation between willingness to use PILBOX and frequency of prescription refills or collection of medications at the pharmacy (116/222, *r*=.05, *P*=.63) among patients who collected their medications in installments. Participant willingness to use PILBOX correlated weakly and negatively with traveling time to the polyclinics where the medication locker stations were located (168/222, *r*=–.14, *P*=.07).

## Discussion

### Principal Findings

This study found that about 40.0% of patients and caregivers were willing to use PILBOX to collect their medications. This acceptance rate of a service innovation is in line with the natural diffusion of any new products or services into use among a population of users, comprising innovators (2.5%), early adopters (13.5%), early majority (34.0%), late majority (34.0%), and laggards (16.0%) [16].

### Willingness to Use PILBOX

The ease of use of PILBOX was the most important factor affecting the desirability of the new medication collection service to users (patients and caregivers). A possible explanation for this observation was that ease of use could elicit positive emotions associated with the use of PILBOX and lead to service satisfaction [17]. Hence, ease of use of PILBOX would intuitively encourage users to opt for the service [18]. As mentioned in the Results section, participants who were not keen to use PILBOX were hesitant because they were worried they would not be able to understand the directions of use as indicated on the locker stations [19], whether because of literacy limitations or poor eyesight. Some participants also provided feedback that they felt they would need assistance to guide them with use of PILBOX while others were put off because they felt they were not tech savvy enough. Further, as seen in Table 3, willingness to use PILBOX increased with education (*P*>.99) and literacy levels (*P*<.001) and decreased with age (*P*=.01). The working status of participants also influenced whether participants were willing to use PILBOX (*P*=.01), with working individuals being more willing to use the service presumably due to their greater exposure to and hence greater confidence in interacting with innovative products and services [19]. Participants living in private housing (versus public housing) and presumably deemed more affluent were also more willing to use PILBOX (*P*=.08), again likely due to their greater exposure to and confidence in interacting with innovative products and services [20]. From the survey results, if greater ease of use such as by providing clear and understandable instructions [21] is designed into PILBOX, there is a higher chance that it will be embraced by more users.

The second most important factor affecting user choice was the zero waiting time advantage offered by the PILBOX option. Previous studies have shown that patient satisfaction levels would reduce with increase in waiting time [22,23]. We would intuitively expect that decreased satisfaction levels on the part of patients would then motivate them to use PILBOX, which could potentially offer them the advantage of zero waiting time, since they could choose when to collect their medications.

Surprisingly, our data showed that user willingness to use PILBOX correlated weakly and negatively with waiting time at the pharmacy (180/222, *r*=–.23, *P*<.001), which was not what we would intuitively expect. This could be due to users having become accustomed to longer waiting times at the polyclinics and having realistic expectations given that they were receiving subsidized medical care. In view of these data, it was uncertain whether the zero waiting time advantage would be a factor to induce users to use the PILBOX service. However, in view that many studies have shown negative association between increased waiting time and patient satisfaction [24,25], no wait time could still be a value proposition for the use of PILBOX.

On the other hand, our results showed that patient willingness to use PILBOX correlated negatively and very weakly with patient satisfaction levels over waiting time at the pharmacies (180/222, *r*=–.06, *P*=.47). This was to be expected, given that a patient who was already satisfied with the pharmacy waiting times would be less motivated to use an alternative mode of medication collection (ie, PILBOX) [26].

The third most important factor affecting patient choice was the cost of using the medication pickup service. Due to price elasticity of demand [27], it was intuitive to expect that the need to pay for use of PILBOX would deter users from opting to use this innovative service [20]. This was borne out by our data. From Table 3, users who were working or had income were more willing to use and pay for the PILBOX service (159/222, *r*=.23, *P*<.001).

The fourth most important factor affecting patient choice was the 24/7 convenience for collection of medications offered to patients by the PILBOX service. This service would be expected to appeal to users as it would allow them to collect their medications at a time convenient to them. Our data showed that there was positive correlation between willingness to use PILBOX and frequency of prescription refills or collection of medications (ie, patients who had to endure more trips to collect their medications would be more willing to explore use of PILBOX to derive greater convenience [116/222, *r*=.05, *P*=.63]).

The least important factor affecting patient choice was the location of PILBOX. Previous studies reported mixed findings on the impact of the traveling distance to a pharmacy or dispensary on medication adherence [28,29]. Some studies reported some association between traveling distance with motivation of patients to collect and adhere to administration/dosing of their medications, whereas other studies reported minimal association [28,29]. Our result showed that willingness to use PILBOX correlated negatively and weakly with the traveling time of users for collection of medications (168/222, *r*=–.14, *P*=.07). This was consistent with previous studies in that the relation between traveling distance and willingness to invest effort to collect prescribed medications was not strong. The weak negative correlation between traveling distance and willingness to use PILBOX can be attributed to PILBOX being located at the polyclinics. Hence, while it provided greater convenience over the medication collection time, patients would still need to travel to the polyclinics to collect their medications. Given the results, there are opportunities to evolve additional options for collection of medications from locations other than polyclinics themselves. This may entail collaboration with suitable partners operating within community hubs located near the residences of patients.

### Remodeling the Medication Collection Process

The rapid development of Singapore into a smart nation and advances in IT capabilities disrupting many industries (eg, transport, food and beverage, and retail industries) have also changed the way health care providers interact with patients and their caregivers [30].

Specifically, in the area of supply of prescribed medications to patients, the conventional norm was to supply prescribed medications to patients face-to-face at pharmacy counters. In the high-patient volume environment in polyclinics [31], pharmacy staff face many challenges in providing excellent service to patients (ie, high patient volumes and prescription loads and long prescriptions with many items due to an aging population) [32-34], high expectations of a more educated patient population, and the need to fulfill patient waiting time targets [35].

Due to the high patient volume and limited space at polyclinics, one problem faced by pharmacy staff was congestion in the pharmacy waiting areas, particularly during peak hours [24]. Instead of instinctive solutions such as increasing staff numbers, which would push up costs and reduce productivity, or pressurizing staff into rushing through the dispensing process, which could compromise medication safety [36], pharmacy staff tried to think outside of the box in coming up with a solution. Breaking from the paradigm that all patients with prescribed medications had to be served within the polyclinic operating hours, pharmacy staff started to think of how some patients could be served asynchronously outside of polyclinic operating hours [37] (eg, having the medications delivered to them or being given an option to collect their medications after their polyclinic visits). From the latter idea, the staff evolved the idea of a medication pick-up service that was gradually developed and eventually fleshed out into the first-generation PILBOX.

One concern was whether this concept of medication pickup would be embraced by the patient population at large. Hence, we undertook this research study to determine the willingness by patients and caregivers to use this new innovation and find out what factors would affect their willingness to use the innovative service. The research findings provided key insights to develop the first generation PILBOX and would guide the pharmacy team to further streamline the PILBOX innovation to better meet the needs of the users so as to increase uptake of the service and enhance pharmacy overall medication supply service to patients.

### Translating Research Findings Into Practice

From the study, the ease of use of PILBOX was a top concern of patients and their caregivers. Hence, when developing the first generation PILBOX service, considerable efforts were devoted to design ease of use into the innovation and assist users with service ambassadors and educational materials [38]. Pictogram decals were pasted on the locker station to provide easy reference to users on use of PILBOX. Educational materials were written at primary 6 level and below and care was taken to ensure that the message content, typography, and visuals of educational materials were designed to promote ease of use [39,40].

To further enhance PILBOX and improve uptake of the innovative service, user inputs will continue to be solicited, either via surveys or focus group discussions [41]. Efforts to educate users on this medication pickup service will continue to be undertaken so that they can confidently use the service [38].

Another finding from the study was that users were more likely to use PILBOX if they did not have to travel too far. Hence, besides giving patients the option of collecting their prescribed medications from PILBOX located within polyclinics, it may be worthwhile for the polyclinics to explore working with suitable partners operating within community hubs located near the residences of patients to make available to users the option of collecting their prescribed medications from locations nearer their homes [19,24].

### Future Generations of PILBOX

PILBOX is a new innovation in Singapore that provides a solution to enable patients to pick up their medications at their convenience 24/7. This reduces the need for patients to wait at home to receive their medications via medication delivery service. The waiting time can then be diverted to better uses, translating into better value and experience for the patient. Further, this alternative mode of medication supply enables more efficient use of limited health care resources that every country faces, including Singapore. This modality of supplying medications can still be considered innovative, as the predominant mode of supplying medications to patients is still face-to-face.

Although automated locker stations may be common nowadays, they may not be suitable for medications requiring special considerations (eg, storage, security and compliance with legal requirements). The unique design of PILBOX enables maintenance of ambient temperature at 25 ℃ (77 ℉) and below. This is imperative as medications are required to be stored within the manufacturer’s recommended storage temperatures to maintain their safety and integrity. Further, PILBOX’s autolock mechanism and lock-down system keep the medication parcel safe and ensure public health safety by preventing unauthorized access without 24/7 human supervision or intervention.

Although the current PILBOX model was well received by patients and their caregivers, future models could include self-cooling features so the locker stations need not be placed only in enclosed locations with 24/7 air conditioning to maintain the required ambient temperatures. The future PILBOX could also have self-refrigerating system to provide appropriate storage condition for cold-chain items such as insulins. This would allow the PILBOX option to be extended to patients prescribed thermolabile medications.

Currently, only pharmacy users are able to book the lockers in PILBOX for use via a designated online portal. Future PILBOX models and IT enablers could provide patients or their caregivers and pharmacy operators with even greater convenience by allowing submission of prescription orders via a mobile app, translation of prescription orders into the pharmacy’s system, picking and packing of medications by robots, and automatic placement of picked/packed medications into medication locker stations for collection by the users. The same mobile app could also provide medication use instructions and advice for reference by users [42,43].

### Limitations of Study

Currently, patients or caregivers can visit private dispensaries or pharmacies and hospital pharmacies in addition to polyclinic pharmacies to fill or refill their prescriptions. A limitation of this study is it was confined to patients and caregivers who visited polyclinics to refill their prescriptions or collect their medications. The results would have been more representative of the population if patients or caregivers who visited private dispensaries or pharmacies and hospital pharmacies had been included in the study. However, this limitation is not of great concern as the proportion of patients who visited polyclinics is significant and it would be reasonable and feasible to extrapolate the findings to the entire local population. Another limitation of this study is that only a pictorial PILBOX prototype was shown to participants during the study. The artist impression and features of PILBOX and the medication collection process involving the PILBOX might have been interpreted or imagined differently by different individuals, thereby potentially creating a visual bias.

### Future Studies

With more people becoming digitally savvy and better technology providing an even more seamless experience for the user, we would expect a higher adoption rate for the use of PILBOX. Further, with people now spending more time at work or having other competing priorities such as caring for family, the convenience provided by PILBOX may also entice more to use this service and even to pay to use it. However, we are mindful that there will always be a segment of the population who will not be comfortable with this new modality of supply as they may not be digitally savvy or may just be simply averse to use of technology. They tend to be older patients who incidentally are more significant because they tend to have more medical conditions and consume more medications. Hence, while this technology is innovative and bring benefits to patients, it may require a period of time running into years for widespread subscription to its use. This study has provided a baseline that future studies could use and compare with to determine the change in user expectations and their willingness to adopt more advanced technology or services.

### Conclusions

A significant proportion of patients and caregivers are keen to use PILBOX as an alternative mode to collect their medications. Addressing patient concerns such as ease of use and language literacy barriers with regard to PILBOX use will help to increase their adoption of this new service.

## Appendix

Multimedia Appendix 1 Questionnaire.

## Abbreviations

IT: information technology
PILBOX: Prescription in Locker Box
