# Supplementary material for: Remodeling the Medication Collection Process With Prescription in Locker Box (PILBOX): Prospective Cross-sectional Study
Source: J Med Internet Res. 2022 Jun 27;24(6):e23266. doi: 10.2196/23266 (PMC9274397; doi:10.2196/23266)
Supplement: Multimedia Appendix 1 [file jmir_v24i6e23266_app1.docx]

**PILBOX Survey**

1. Do you normally collect your regular medications in instalments?

- Yes
- No

If Yes, how often do you collect the instalments?

- More often than once every month (please indicate ___________)
- Once every month
- Once every 3 months
- Once every 6 months
- Others: ________________

2. On average, how long do you need to wait at the polyclinic pharmacy before you get your medications?

- Less than 15 minutes
- Between 15-30 minutes
- Between 30-45 minutes
- Between 45-60 minutes
- More than 60 minutes

3. On a scale of 1 to 5, please indicate your level of satisfaction with regards to the average waiting time. (1 being **least** satisfied and 5 being **most** satisfied)

1 2 3 4 5

_____________________________

Least satisfied Most satisfied

4. On a scale of 1 to 5, please indicate your willingness to use a self-service medication locker station located at the Polyclinic for the collection of your refill (or instalment) medications, instead of collecting at the pharmacy (assuming no cost is involved at all)?

(1 being not keen at all and 5 being very keen)

1 2 3 4 5

_____________________________

Not keen Neutral Very keen

Please provide your reason(s):

__________________________________________________________________________________________________________________________________________________________________________

5. If this self-collection service offers you the advantages of picking up your refill (or instalment) medications at your convenience and without having to wait, how much would you be willing to pay per collection?

- Less than $2
- Between $2-$5
- Between $5-10
- More than $10
- Not willing to pay at all
  - If so, please indicate the reason(s) why:

________________________________________________________________________________________________________________________________________________

6. Please rank the following factors that will affect your decision to use the self-service medication locker station (1 being the **least** important and 5 being the **most** important)

___ Accessible 24/7

___ No waiting tim

___ Ease of use

___ Cost of use

___ Location of the station

Others, please specify:

__________________________________________________________________________________________________________________________________________________________________________
